# Serum leptin is associated with increased pulse pressure and the development of arterial stiffening in adult men: results of an eight-year follow-up study

**DOI:** 10.1038/s41440-021-00718-x

**Published:** 2021-08-12

**Authors:** Lanfranco D’Elia, Alfonso Giaquinto, Roberto Iacone, Ornella Russo, Pasquale Strazzullo, Ferruccio Galletti

**Affiliations:** Department of Clinical Medicine and Surgery, ESH Excellence Center of Hypertension, “Federico II” University of Naples Medical School, Naples, Italy

**Keywords:** Adipocytokines, Adipokines, Arterial stiffening, Leptin, Pulse pressure

## Abstract

High leptin levels are associated with an unfavorable cardiometabolic risk profile. A number of studies found a positive association between leptin and vascular damage, but to date, no observational study has evaluated a potential predictive role of leptin for arterial stiffening. Therefore, the aim of this study was to estimate the role of leptin in the incidence of arterial stiffening (pulse pressure >60 mmHg) and changes in pulse pressure in an 8-year follow-up of a sample of adult men (The Olivetti Heart Study). The analysis included 460 men without baseline arterial stiffening and antihypertensive treatment at baseline and at follow-up (age: 50.0 years, BMI: 26.5 kg/m^2^). At the end of the follow-up period, the incidence of arterial stiffening was 8%. Baseline leptin was significantly greater in the group that developed arterial stiffening and was significantly correlated with pulse pressure changes over time (*p* < 0.05). According to the median plasma leptin distribution of the whole population, the sample was stratified into two groups: one with leptin levels above the median and the other with leptin levels below the median. Those who had baseline leptin levels above the median had a greater risk of developing arterial stiffening (odds ratio: 2.5, *p* < 0.05) and a greater increase in pulse pressure over time (beta: 2.1, *p* < 0.05), also after adjustment for confounders. The results of this prospective study indicate a predictive role of circulating leptin levels for vascular damage, independent of body weight and blood pressure.

## Introduction

Leptin, a hormone mainly produced by adipocytes, plays a principal role in the regulation of body weight [1]. However, individuals with excess body weight have high leptin levels, thus suggesting ineffective metabolic actions of leptin, namely, leptin resistance [2]. Indeed, a number of studies detected an unfavorable association between high leptin levels and cardiometabolic risk profiles, mostly in obese subjects [3]. On the other hand, our previous investigations showed that leptin was positively associated with cardiovascular risk independent of body weight, increasing the risk of hypertension [4] and insulin resistance [5] and decreasing renal function [6]. In addition, leptin seems to be involved in inflammation [7] and in the modulation of uric acid excretion and hence circulating uric acid levels [8].

In this context, some studies were carried out to evaluate the association between leptin and asymptomatic cardiovascular organ damage, such as arterial stiffening (AS), which is associated with increased cardiovascular risk [9]. AS can be assessed by different noninvasive measurement methods, of which carotid-femoral pulse wave velocity (cfPWV) (the gold standard method) and pulse pressure (PP) are officially recognized [9].

Experimental evidence found that leptin can adversely affect vascular function by stimulating the renin–angiotensin–aldosterone system (RAAS) [10], impairing sodium handling [11], increasing sympathetic nerve activity [12] and reactive oxygen species formation [13], and promoting endothelial cell growth [14]. In addition, a number of observational studies analyzed the association between leptin and vascular damage, but these studies were cross-sectional. Most of them found a positive association between leptin and AS both in patients with cardiovascular risk (e.g., chronic kidney disease, coronary heart disease, diabetes, hypertension) and in the general population [15]. However, few observational investigations found an inverse relationship in young and relatively lean participants [16, 17].

Considering that no prospective data were available on the predictive role of leptin for AS, the aim of our study was to prospectively analyze baseline leptin levels in relation to the development of AS in a sample of men participating in the Olivetti Heart Study (OHS).

## Methods

### Study population

The OHS was an occupational investigation of the male workforce of the Olivetti factories in southern Italy (Pozzuoli-Naples and Marcianise-Caserta) and has been previously described [18, 19].

The Ethics Committee of “Federico II” University in Naples approved the Olivetti study protocol, and the participants provided their informed written consent to participate.

This study was planned, conducted, and reported according to the STROBE statement (https://www.equator-network.org/reporting-guidelines/strobe/) (Supplemental Table).

A total of 1085 individuals (95% of the total male workforce) aged 25–75 years were examined in 1994–95, and 84% were seen again in 2002–04. For the purposes of the present analysis, we sequentially excluded (1) participants whose demographic and anthropometric characteristics and cardiometabolic risk factors were not available at baseline and at follow-up (*n* = 290); (2) participants who used antihypertensive therapy at baseline and at follow-up (*n* = 309); and (3) participants with AS at baseline (*n* = 16). Finally, the evaluation of AS risk was performed on 460 participants.

### Examination procedures

The OHS study procedures have been described previously [18, 19]. Briefly, physical examinations at both baseline and follow-up visits were performed between 08:00 and 11:00 h, with the participants having fasted for at least 13 h. Baseline and 8-year follow-up visits included a physical examination, anthropometric measurements, a blood test, and the administration of a questionnaire.

Systolic and diastolic blood pressure (BP) (phase V) were measured three times at 2-min intervals with a random zero sphygmomanometer (Gelman Hawksley Ltd., Sussex, UK) after the subject had been sitting for at least 10 min. The average of the second and third readings was recorded. PP was calculated by the following formula: systolic BP minus diastolic BP. A PP >60 mmHg was considered a cutoff value for AS [9].

The questionnaire classified the participants into current smokers, never smokers, and ex-smokers. Physical activity level was expressed according to whether the participant habitually engaged in at least 30 min per day of aerobic exercise (yes/no). Participants were also classified into two groups according to their alcohol intake: at least one glass of wine (or an equivalent amount of other alcoholic beverages per day) (yes) or no alcohol consumption (no).

A fasting venous blood sample was taken with the participants in the seated position. Blood specimens were immediately centrifuged and stored at −70 °C until analysis. Serum leptin was measured by an enzyme-linked immunosorbent assay (R&D System GmbH, Wiesbaden-Nordenstadt, Germany). Serum creatinine was measured by the picric acid colorimetric method. The estimated glomerular filtration rate (eGFR) was estimated by a standard formula [20]. Serum glucose levels were measured with automated methods (Cobas-Mira, Roche, Italy). Serum insulin was determined by radioimmunoassay (Insulin Lisophase; Technogenetics, Milan, Italy). Insulin sensitivity was estimated by the homeostasis model assessment (HOMA index) using the formula fasting plasma insulin (μU/mL) × fasting plasma glucose (mmol/L)/22.5. A HOMA index >2.77 UI was considered a cutoff value for insulin resistance. Body weight and height were measured on a standard beam balance scale with an attached ruler. Body mass index (BMI) was measured according to the formula weight (kg)/height^2^ (m). Excess body weight was defined as a BMI ≥25 kg/m^2^. Waist circumference (WC), an indicator of abdominal adiposity, was measured at the level of the umbilicus with the subject standing erect with flexible inextensible plastic tape. Abdominal obesity was defined as a WC >102 cm.

### Statistical analysis

All statistical analyses were performed using SPSS software, version 23 (SPSS Inc., Chicago, IL).

Because baseline leptin, HOMA index, eGFR, and PP values had a skewed distribution, nonparametric tests were used for unadjusted analyses, while after log-transformation, parametric tests were used for analyses adjusted for confounders, except for PP, which was transformed into rank to be included as a covariate in the multivariate analysis of AS risk. Changes in the participants’ main characteristics were calculated as final minus basal measurements.

The participants were stratified into groups of subjects who developed AS (AS[+]) and subjects who did not develop AS (AS[−]) and into quartiles of changes in PP (median, first tertile (I-T): −9.9 mmHg, second tertile (II-T): −0.4 mmHg, third tertile (III-T): 7.4 mmHg, fourth tertile (IV-T): 18.3 mmHg). In addition, the participants were also stratified according to the median plasma leptin distribution of the whole OHS population (2.97 ng/mL) into groups of subjects with low (leptin[−]) and subjects with high leptin (leptin[+]) levels.

Bivariate relationships between leptin and the variables under investigation were evaluated by Spearman’s correlation analysis. A multivariable linear regression analysis was carried out to determine the independent effect of baseline (continuous and categorical) leptin on changes in PP, adjusting for the main potential confounders.

To evaluate significant differences between groups (i.e., AS[+] vs. AS[−]), analysis of variance or the Mann–Whitney U test was used. The chi-squared test was used to evaluate differences between categorical variables. Binary logistic regression analysis was used to estimate the role of leptin (leptin[+] vs. leptin[−] or continuous values) on the development of AS, adjusting for the main potential confounders. Given the strong relationship between BMI and WC (*r* = 0.80, *p* < 0.01), multivariate analyses were separately adjusted for BMI or WC.

The results are reported as the mean with standard deviation, as a percentage, or as an odds ratio (OR) and 95% confidence interval (95% CI), unless otherwise indicated. Two-sided P values below 0.05 were considered statistically significant.

## Results

The baseline characteristics of the whole sample (*n* = 460) are reported in Table 1. The mean age was 50.0 years; 70% of participants had excess body weight, 13% had abdominal obesity, and 19% had insulin resistance. In addition, 45% of participants were smokers, 33% habitually engaged in aerobic exercise, and 84% consumed alcohol.

**Table 1:** Baseline characteristics of the total study participants, and stratified by the development of arterial stiffening at follow-up

	Total	[AS−]	[AS+]
No. of participants	460	422	38
Age (years)	50.0 (7.1)	49.5 (7.0)	54.6 (5.4)^a^
BMI (kg/m^2^)	26.5 (3.0)	26.4 (2.9)	27.6 (3.6)^a^
Waist Circumference (cm)	93.3 (8.3)	93.0 (8.2)	96.5 (8.5)^a^
Systolic BP (mmHg)	121.8 (11.9)	121.0 (11.7)	130.6 (11.2)^a^
Diastolic BP (mmHg)	80.5 (8.2)	80.0 (7.8)	84.9 (10.9)^a^
eGFR (mL/min/1.73 m^2^)^b^	97.7 (1.2)	97.7 (1.2)	95.5 (1.1)^c^
PP (mmHg)^b^	40.7 (1.2)	39.8 (1.2)	45.2 (1.1)^a, c^
HOMA index (Unit)^b,d^	1.8 (1.7)	1.8 (1.7)	2.1 (1.9)^c^
LPT (ng/mL)^b^	2.5 (2.2)	2.4 (2.2)	3.2 (2.4)^a, c^

Data are expressed as means (SD) or as percentages

*AS* arterial stiffening at follow-up, *BMI* body mass index, *BP* blood pressure, *eGFR* estimated glomerular filtration rate, *HOMA* homeostatic model assessment, *LPT* leptin, *PP* pulse pressure

^a^AS[+] vs. AS[−]: *p* < 0.05

^b^Data expressed as geometric mean

^c^Analysis performed by the Mann–Whitney U test

^d^*n* = 440

The analysis of the correlation between leptin levels and the most relevant characteristics of participants at baseline showed that leptin levels had a significant and positive association with BMI (*r* = 0.56, *p* < 0.01), WC (*r* = 0.56, *p* < 0.01), BP (systolic BP: *r* = 0.20, *p* < 0.01; diastolic BP: *r* = 0.25, *p* < 0.01) and HOMA index (*r* = 0.27, *p* < 0.01) but not with age, renal function or PP (*p* > 0.05).

At the 8-year follow-up, an overall incidence of AS of 8% was detected. Table 1 shows the differences in baseline features between AS[+] and AS[−] participants at follow-up. The AS[+] group had higher age, BMI, and WC than the AS[−] group. In addition, baseline systolic and diastolic BP, as well as PP, were significantly higher in AS[+] than in AS[−] participants. Likewise, the percentage of AS[+] participants was significantly higher among smokers than among nonsmokers (13% vs. 4%, *p* < 0.05) and among those who habitually engaged in aerobic exercise than among those who did not (15% vs. 5%, *p* < 0.05). In contrast, no difference was detected in renal function, insulin sensitivity, or alcohol consumption.

**Fig. 1:**
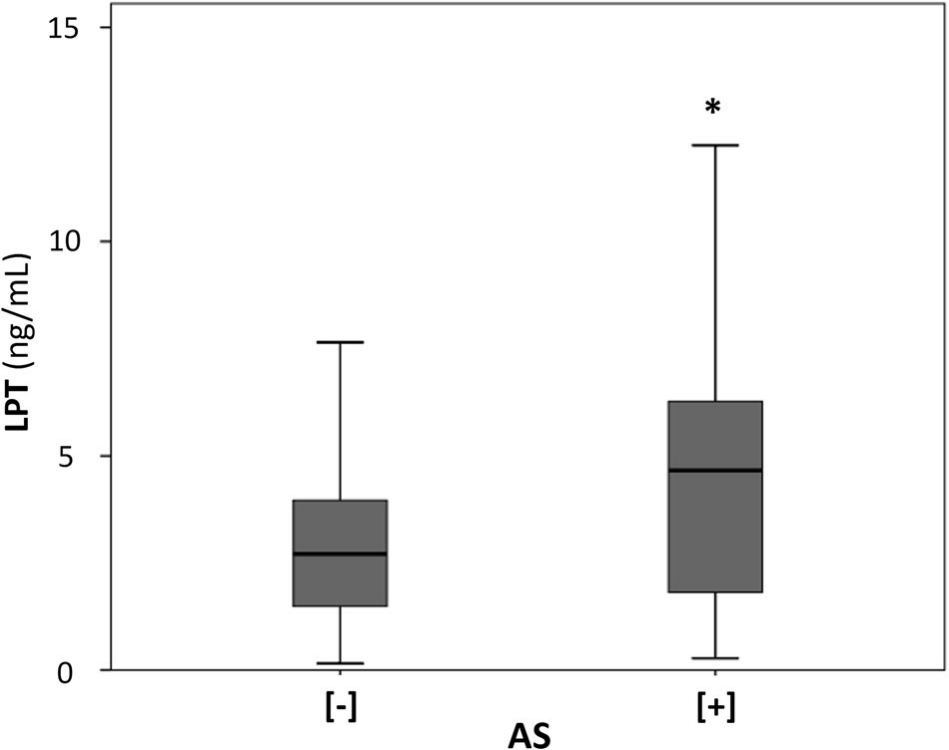
Baseline leptin (LPT) levels and development of arterial stiffening (AS). AS[−]: no arterial stiffening, AS[+]: arterial stiffening. LPT data expressed as geometric mean (ng/mL). Black line in the boxes: median value. *p < 0.01

Leptin levels were significantly higher in AS[+] participants than in AS[−] participants (Fig. 1) and were significantly correlated with PP changes over time (*r* = 0.11, *p* = 0.02). The positive correlation between baseline leptin and PP changes over time was also confirmed in the linear regression analysis adjusted for baseline age, PP value, excess body weight, renal function, smoking, physical activity, alcohol consumption, hypolipidemic therapy and insulin resistance (Table 2). The results, including changes in body weight over the years, were not substantially modified in the models (Table 2). In addition, the predictive role of leptin was unchanged when WC or its changes over time was included.

**Table 2:** Baseline leptin and changes in pulse pressure over time

	Changes in PP (Dependent variable) Beta (95% CI)	*P* value
Leptin[+] vs. Leptin[−] ^a^ (Independent variable)		
Unadjusted	3.85 (2.52–5.18)	<0.001
Multivariable Model a^b^	2.10 (0.38–3.82)	0.017
Multivariable Model b^c^	2.15 (0.35–3.95)	0.019
Multivariable Model c^d^	2.65 (1.00–4.29)	0.002
Multivariable Model d^e^	2.57 (0.84–4.31)	0.004
1-SD increase in log-Leptin^f^ (Independent variable)		
Unadjusted	1.03 (0.06–1.98)	0.03
Multivariable Model a^b^	1.09 (0.21–1.97)	0.01
Multivariable Model b^c^	1.07 (0.17–1.98)	0.02
Multivariable Model c^d^	1.39 (0.57–2.21)	0.001
Multivariable Model d^e^	1.32 (0.47–2.17)	0.002

*PP* pulse pressure

^a^Baseline Leptin[+] (Leptin > 2.97 ng/mL) vs. baseline Leptin[−] (Leptin < 2.97 ng/mL)

^b^Adjusted for baseline age, pulse pressure (rank), body weight, renal function (log-transformed eGFR), smoke, physical activity, and alcohol consumption

^c^Adjusted for Model a plus Insulin resistance and hypolipidemic therapy at baseline (*n* = 440)

^d^Adjusted for Model a plus body weight changes over time instead of body weight at baseline

^e^Adjusted for Model b plus body weight changes over time instead of body weight at baseline (*n* = 440)

^f^1-SD log-Leptin = 2.2 ng/mL

**Fig. 2:**
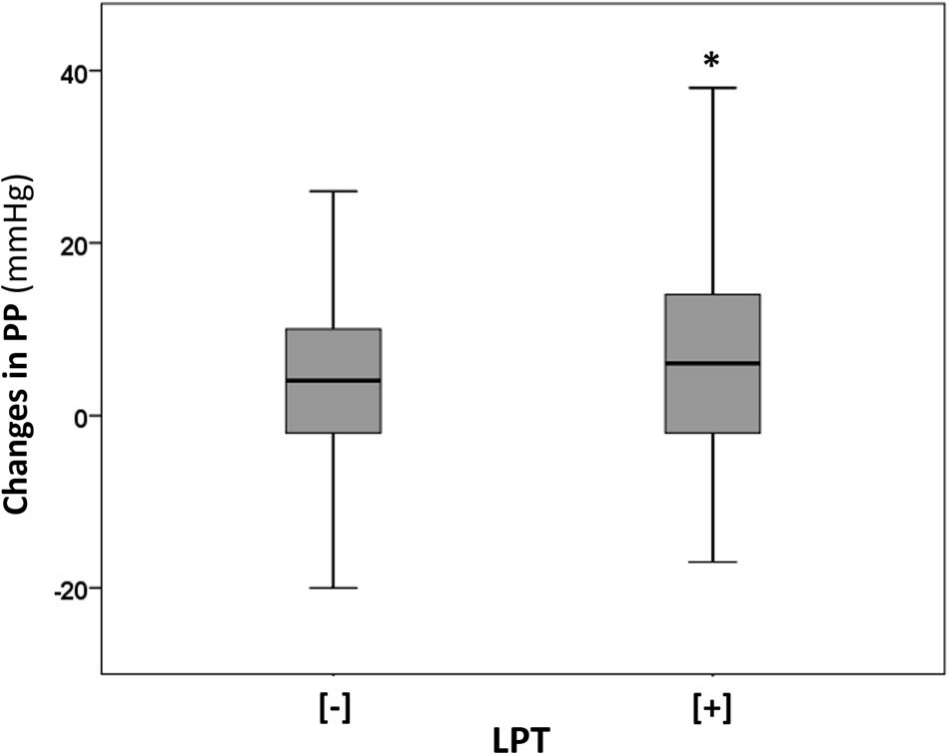
Changes in pulse pressure (PP) over time and baseline leptin (LPT) stratified by median levels. LPT[−]: leptin below the median, LPT[+]: leptin above the median. Black line in the boxes: median value. *p = 0.01

A similar association was found when baseline leptin was stratified by the median value. Indeed, changes in PP over time were significantly greater in the leptin[+] group than in the leptin[−] group (Fig. 2), which was also confirmed after adjustment for the same main confounders (Table 2).

The percentage of participants who developed AS was greater in the leptin[+] group than in the leptin[−] group (11% vs. 5%, *p* = 0.02). The logistic regression analysis confirmed the significant positive association between baseline leptin and the development of AS over time (leptin[+] vs. leptin[−], OR: 2.2; 95% CI: 1.1–4.4) (Table 3). The association remained statistically significant after accounting for baseline age, PP value, excess body weight, renal function, smoking, physical activity, alcohol consumption, hypolipidemic therapy, and insulin resistance (Table 3). Similar results were detected when central obesity was included in the models instead of body weight (OR: 2.57, 95% CI: 1.12–5.87). Moreover, we carried out analyses including changes in body weight or WC over the years as covariates. In addition, these models showed a significant predictive role of leptin for AS (model with changes in BMI, OR: 2.92, 95% CI: 1.32–6.43; model with changes in WC, OR: 2.86, 95% CI: 1.28–6.37). In addition, adjusting for changes in (systolic or diastolic) BP over time, the results were confirmed (systolic BP changes, OR: 2.70, 95% CI: 1.07–6.84; diastolic BP changes, OR: 2.39, 95% CI: 1.05–5.44).

**Table 3:** Eight-year risk of incident arterial stiffening for greater baseline leptin levels

	Risk of AS (Dependent variable) OR (95% CI)	*P* value
Leptin[+] vs. Leptin[−]^a^ (Independent variable)		
Unadjusted	2.22 (1.10–4.45)	0.025
Multivariable Model a^b^	2.50 (1.11–5.64)	0.028
Multivariable Model b^c^	2.45 (1.07–5.63)	0.035
Multivariable Model c^d^	3.02 (1.40–6.51)	0.005
Multivariable Model d^e^	2.92 (1.32–6.43)	0.008

*AS* arterial stiffening, *OR* odds ratio

^a^Baseline LPT[+] (Leptin > 2.97 ng/mL) vs. baseline LPT[−] (Leptin < 2.97 ng/mL)

^b^Adjusted for baseline age, pulse pressure (rank), body weight, renal function (log-transformed eGFR), smoke, physical activity, and alcohol consumption

^c^Adjusted for Model a plus Insulin resistance and hypolipidemic therapy at baseline (*n* = 440)

^d^Adjusted for Model a plus body weight changes over time instead of body weight at baseline

^e^Adjusted for Model b plus body weight changes over time instead of body weight at baseline (*n* = 440)

We also explored the role of leptin as a continuous variable in AS risk. The analysis revealed that a higher baseline leptin value of 2.2 ng/mL (1-SD increase) was associated with a significantly increased risk of developing AS (OR: 1.5, 95% CI: 1.1–2.3). The risk did not substantially change after accounting for the main potential confounders (*p* < 0.05).

Finally, we carried out separate analyses after stratification by quartiles of changes in PP. The percentage of leptin[+] participants linearly increased across PP-change quartiles (I-T: 18.1%, II-T: 22.2%, III-T: 26.7%, IV-T: 33.0 *p* = 0.007). In addition, multivariate analysis confirmed the positive predictive role of leptin on PP changes after adjusting for the main confounders (*p* < 0.05).

## Discussion

To our knowledge, this is the first study directly relating baseline leptin levels to the risk of AS and changes in PP values over time in a relatively large middle-aged sample selected from a general population observed for a reasonably long period. In particular, our results indicate that the predictive role of leptin levels both as continuous values and stratified by threshold level on AS is independent of potential confounders, such as age, insulin sensitivity, anthropometric indices, and BP at baseline, and changes in anthropometric measures and BP over time. In addition, a similar role of baseline leptin was found when changes in PP over time were explored.

In the absence of a threshold for circulating leptin, our results suggest that leptin values greater than 2.9 ng/mL are associated with a twofold increased risk of developing AS, independent of body weight and BP, which is in line with our previous studies [3–6, 8].

As previously found [6], this analysis did not detect any association between leptin and renal function at baseline or between leptin and PP, which was likely due to the sample included in the analysis (participants who were not on antihypertensive therapy at baseline and at follow-up, who did not have arterial stiffening at baseline and who were relatively young).

Existing experimental data support this relationship. Indeed, a large body of evidence indicates that leptin stimulates RAAS [10], increases reactive oxygen species formation [13], reduces nitric oxide bioavailability [21], regulates myocardial endothelin-1 expression [22], promotes endothelial cell growth [14], and induces cardiovascular smooth muscle cell proliferation [23]. Leptin may exert an effect on cardiovascular organ damage, increase sympathetic nerve activity, bind to specific receptors in the central nervous system and in the kidney [24], or impair sodium handling [11, 25]. Furthermore, the positive relationship of leptin with insulin resistance [5] and uric acid levels [8] and the inverse relationship with renal function [6] may contribute to this vascular damage [25].

In addition, observational studies confirmed the association between leptin and AS, particularly with cfPWV; however, no studies prospectively evaluated the AS risk [15]. The majority of studies detected a positive association between leptin and AS, including in patients with chronic kidney disease, diabetes, coronary heart disease, or hypertension [15]. This positive relationship was also found both in a multiethnic cohort and in a large European general population [15]. In contrast, there was an inverse relationship between leptin and AS in two large samples of general relatively lean young participants [16, 17]. This inverse association may be due to the absence of leptin resistance in these cohorts.

On the other hand, some classes of drugs could affect the predictive role of leptin both by modulating leptin levels and interacting with BP-linked organ damage, which in part would explain its favorable effect on cardiovascular risk [26–28]. Of note, based on such evidence, leptin levels and their association with the risk of AS in our sample were not affected by medications. Indeed, participants with antihypertensive therapy at baseline and at follow-up were excluded, and no subjects with antidiabetic treatment were included in the sample. Moreover, the logistic models adjusted for hypolipidemic therapy at baseline confirmed the predictive role of leptin.

### Study strengths and limitations

The strengths of our study are (1) the prospective design with the relatively long follow-up period; (2) the large number of included participants from a general population who were not undergoing BP treatment and did not have AS at baseline; (3) the careful standardization of data collection at both baseline and the follow-up examination; (4) the association between (continuous and categorical) leptin and organ damage independent of body weight; and (5) no bias related to any pharmacological treatment.

Nonetheless, the study has some limitations. The first limitation is the participation of only adult white male individuals, which makes our results only generalizable to male Caucasian people. Indeed, there is a difference in leptin levels between men and women [29], and there is likely also a gender or race difference in the influence of leptin on cardiovascular organ damage [30]. Second, the absence of direct assessment of AS may be a limitation of the study. Indeed, PP is influenced both by the compliance of the large arteries and the stroke volume. In particular, stroke volume has a more marked influence on PP in younger men than older men. Hence, the development of AS may not be solely due to the process of arteriosclerosis, especially in younger individuals. However, although there was a wide age range among the individuals included in the analysis, the group that developed AS was older at baseline. Hence, also in consideration of the prospective design of the study, the contribution of the stroke volume could be neglected in this context.

In addition, the designation of AS groups may be a limitation because AS is a continuous age-dependent process. However, although potentially arbitrary, the cutoff of 60 mmHg is supported by a large amount of evidence [9, 31, 32]. Moreover, PP values greater than 60 mmHg indicate AS, as well as cfPWV values greater than 10 m/s, which is the gold standard among AS noninvasive measurement methods [9].

Another limitation is the lack of intermediate parameters measured during the follow-up period, with the consequent inability to perform a time-to-event analysis relative to the development of cardiovascular damage. In addition, the lack of assessment of serum leptin-interacting proteins and circulating leptin receptors may be a limitation. Finally, although we adjusted the models for many important covariates, there is still a possibility of residual confounding.

## Conclusions

The present analysis of a sample of an adult male population indicated that higher baseline leptin levels predict the development of vascular damage expressed as a risk of AS and changes in PP, independent of the main potential confounders. These results were supported by the predictive role of leptin found in both the quantitative and qualitative analyses.

Considering that PP greater than 60 mmHg is an important cardiovascular risk factor [9, 31, 32], the association detected in this study significantly adds to the recognized value of leptin in cardiovascular risk accumulated through previous studies, and in addition, it suggests a role of early markers to predict cardiovascular disease.

Given the prominent role of AS in cardiovascular risk, further studies are needed to support our conclusions and to better clarify the mechanisms involved.

## Supplementary information

Supplementary Table
